# Effectiveness of a Smartphone App to Promote Healthy Weight Gain, Diet, and Physical Activity During Pregnancy (HealthyMoms): Randomized Controlled Trial

**DOI:** 10.2196/26091

**Published:** 2021-03-11

**Authors:** Johanna Sandborg, Emmie Söderström, Pontus Henriksson, Marcus Bendtsen, Maria Henström, Marja H Leppänen, Ralph Maddison, Jairo H Migueles, Marie Blomberg, Marie Löf

**Affiliations:** 1 Department of Biosciences and Nutrition Karolinska Institutet Huddinge Sweden; 2 Department of Health, Medicine and Caring Sciences Linköping University Linköping Sweden; 3 Folkhälsan Research Center Helsinki Finland; 4 Faculty of Medicine University of Helsinki Helsinki Finland; 5 Institute for Physical Activity and Nutrition Deakin University Burwood Australia; 6 PROFITH "PROmoting FITness and Health through physical activity" research group, Department of Physical Education and Sports, Faculty of Sport Sciences, Research Institute of Sport and Health University of Granada Granada Spain; 7 Department of Obstetrics and Gynecology Linköping University Linköping Sweden; 8 Department of Biomedical and Clinical Sciences Linköping University Linköping Sweden

**Keywords:** gestational weight gain, physical activity, diet, pregnancy, mHealth, smartphone app, mobile phone app, telemedicine, randomized controlled trial

## Abstract

**Background:**

Excessive gestational weight gain (GWG) during pregnancy is a major public health concern associated with negative health outcomes for both mother and child. Scalable interventions are needed, and digital interventions have the potential to reach many women and promote healthy GWG. Most previous studies of digital interventions have been small pilot studies or have not included women from all BMI categories. We therefore examined the effectiveness of a smartphone app in a large sample (n=305) covering all BMI categories.

**Objective:**

To investigate the effectiveness of a 6-month intervention (the HealthyMoms app) on GWG, body fatness, dietary habits, moderate-to-vigorous physical activity (MVPA), glycemia, and insulin resistance in comparison to standard maternity care.

**Methods:**

A 2-arm parallel randomized controlled trial was conducted. Women in early pregnancy at maternity clinics in Östergötland, Sweden, were recruited. Eligible women who provided written informed consent completed baseline measures, before being randomized in a 1:1 ratio to either an intervention (n=152) or control group (n=153). The control group received standard maternity care while the intervention group received the HealthyMoms smartphone app for 6 months (which includes multiple features, eg, information; push notifications; self-monitoring; and feedback features for GWG, diet, and physical activity) in addition to standard care. Outcome measures were assessed at Linköping University Hospital at baseline (mean 13.9 [SD 0.7] gestational weeks) and follow-up (mean 36.4 [SD 0.4] gestational weeks). The primary outcome was GWG and secondary outcomes were body fatness (Bod Pod), dietary habits (Swedish Healthy Eating Index) using the web-based 3-day dietary record Riksmaten FLEX, MVPA using the ActiGraph wGT3x-BT accelerometer, glycemia, and insulin resistance.

**Results:**

Overall, we found no statistically significant effect on GWG (*P*=.62); however, the data indicate that the effect of the intervention differed by pre-pregnancy BMI, as women with overweight and obesity before pregnancy gained less weight in the intervention group as compared with the control group in the imputed analyses (–1.33 kg; 95% CI –2.92 to 0.26; *P*=.10) and completers-only analyses (–1.67 kg; 95% CI –3.26 to –0.09; *P*=.031]). Bayesian analyses showed that there was a 99% probability of any intervention effect on GWG among women with overweight and obesity, and an 81% probability that this effect was over 1 kg. The intervention group had higher scores for the Swedish Healthy Eating Index at follow-up than the control group (0.27; 95% CI 0.05-0.50; *P*=.017). We observed no statistically significant differences in body fatness, MVPA, glycemia, and insulin resistance between the intervention and control group at follow up (*P*≥.21).

**Conclusions:**

Although we found no overall effect on GWG, our results demonstrate the potential of a smartphone app (HealthyMoms) to promote healthy dietary behaviors as well as to decrease weight gain during pregnancy in women with overweight and obesity.

**Trial Registration:**

ClinicalTrials.gov NCT03298555; https://clinicaltrials.gov/ct2/show/NCT03298555

**International Registered Report Identifier (IRRID):**

RR2-10.2196/13011

## Introduction

### Background

Excessive gestational weight gain (GWG) is a major public health problem [1,2]. In the United States and Europe, around 50% of pregnant women exceed the recommended GWG provided by the National Academy of Medicine [1] with similar data from Sweden [3,4]. Excessive GWG is associated with increased risk of cesarean delivery, gestational diabetes, pre-eclampsia, and obesity in both mother and child [2,5]. Furthermore, previous studies have shown that approximately 20% of American and European women gain less weight than recommended during pregnancy [1], which is associated with complications such as an increased risk of low birth weight and preterm birth [6]. Thus, effective and evidence-based strategies to promote a healthy GWG are of great importance.

Traditional interventions (eg, face-to-face counseling and supervised exercise sessions) to reduce the risk of excessive GWG have been reported to be successful [7-9]. For example, a Cochrane review [9] found that traditional interventions focusing on diet, exercise, or both have been found to reduce the risk of excessive GWG by 20%. Correspondingly, a recent systematic review and meta-analysis [10] found that interventions aiming to improve diet and physical activity behaviors have shown to reduce GWG by 1.81 kg (95% CI –3.47 to –0.16; 21 studies, n=6920) in pregnant women with overweight and obesity [10]. However, traditional interventions are generally resource heavy and rely considerably on health care staff. Furthermore, they are costly and lack the ability to reach large numbers of women.

In the last decade, the use of digital technologies (eg, mobile Health [mHealth]) to deliver lifestyle interventions has increased. In comparison to traditional interventions, mHealth interventions have the advantages of being more cost-effective and accessible [11] and may reduce burden on health care staff. Accumulating evidence indicates that interventions delivered using this technology may promote weight loss [12] and increase physical activity in adults [13]. Furthermore, a recent mHealth pilot study (n=54) in pregnant women with overweight and obesity found that a lower proportion in the intervention group exceeded the recommended GWG compared with the control group who received usual care (58% vs 85%) [14]. However, as highlighted in a recent review [15], mHealth interventions focusing on healthy GWG have generally been pilot studies with small sample sizes (eg, [14,16-18]), or have not included all BMI categories (eg, [19-22]). Moreover, to the best of our knowledge, no previous study has investigated the effectiveness of a behavior change program delivered solely through a smartphone app on GWG in women covering all BMI categories.

### Aim

The aim of this randomized controlled trial was to investigate the effectiveness of the 6-month intervention (the HealthyMoms app) on GWG (primary outcome), body fatness, dietary habits (Swedish Healthy Eating Index), moderate-to-vigorous physical activity (MVPA), glycemia, and insulin resistance (secondary outcomes) in gestational week 37 among Swedish women.

## Methods

### Study Design

The HealthyMoms trial (clinicaltrials.gov NCT03298555) was a 2-arm parallel design randomized controlled trial conducted between October 2017 and November 2020 in the county of Östergötland, Sweden. The study received approval from the Regional Ethical Review Board in Linköping, Sweden (reference numbers 2017/112-31 and 2018/262-32) and all women provided written informed consent before entering the trial. Development of the HealthyMoms app and full details of the study design have been described previously [23]. The study is reported according to the Consolidated Standards of Reporting Trials of Electronic and Mobile Health Applications and online Telehealth (CONSORT-EHEALTH) statement [24] (Multimedia Appendix 1).

### Participants and Procedures

Between October 2017 and March 2020 participants were recruited in early pregnancy at the first routine visit at maternity clinics in the county of Östergötland, Sweden. During the study period approximately 4000 eligible women attended maternity care. At the maternity clinic, participants received written information about the study, and women interested in participating contacted the research team via email or postal mail. Inclusion criteria were aged 18 years or older, a singleton pregnancy, and the ability to read and speak well-enough Swedish to be able to understand the app content. Women previously diagnosed with an eating disorder, diabetes, or other medical conditions with possible effects on body weight were excluded. Eligible women who agreed to participate were sent an accelerometer to assess physical activity and were instructed to register their diet using a web-based dietary assessment tool prior to the measurement. Baseline measures (13.9 [SD 0.7] gestational weeks) and follow-up measures (36.4 [SD 0.4] gestational weeks) were conducted at Linköping University Hospital. In short, these measures included assessment of body weight and height, body composition, plasma glucose, serum insulin, and sociodemographic variables. These are described in more detail below.

### Control Group

The control group received standard maternity care consisting of regular monitoring of maternal and fetal health (such as measurements of blood pressure, blood glucose and ferritin, weight gain, symphysis fundus, as well as fetal movements and heart rate). Standard care also included an optional lecture in early pregnancy on a healthy lifestyle with some brief and general advice on diet, physical activity, smoking and alcohol, pregnancy-related health (eg, nausea, iron deficiency, pelvic pain), and medical care (eg, midwife visits, information on fetal diagnostics). In addition, standard care included repeated measurements of body weight throughout pregnancy.

### Intervention

**Figure 1:**
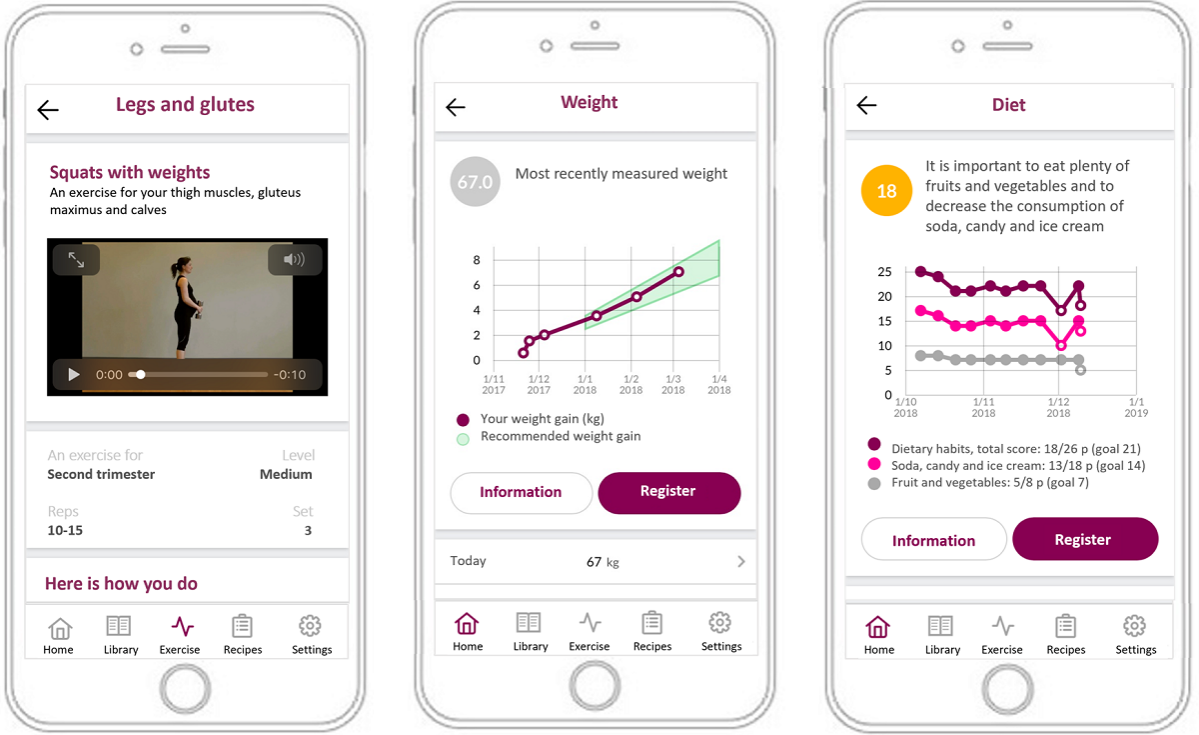
Screenshots from the HealthyMoms app showing examples from the app (ie, an exercise video, the weight gain chart, and diet registration with feedback).

In addition to standard maternity care, participants in the intervention group received the HealthyMoms app (Android and iOS compatible), a 6-month program aimed at promoting recommended GWG [23] by encouraging a healthy diet and physical activity in accordance with current guidelines [25,26]. The app focuses on healthy dietary and physical activity habits as well as healthy GWG, irrespective of pre-pregnancy BMI. Figure 1 shows 3 screenshots from the HealthyMoms app. The development of the HealthyMoms app and its features has previously been described in detail [23]. In short, the app is grounded in social cognitive theory [27] and uses key behavior change techniques (eg, shaping knowledge, goal setting, feedback, and monitoring) which have been suggested to be important for promoting a healthy lifestyle also in pregnant women [15,28-30]. The app consists of 7 features, including informational themes that change every other week, push notifications, self-monitoring with feedback (for diet, physical activity, and GWG), recipes, exercise guide (eg, aerobic and resistance exercises and training programs) and videos, pregnancy calendar, and an app library (eg, frequently asked questions, practical tips). The themes address various topics, such as healthy food choices, exercise during pregnancy, a healthy GWG, and how to change habits. Participants also received automated push notifications 4 times/week with information, support, strategies, and guidance on how to achieve a behavior change and establish or maintain healthy habits (eg, improve diet and increase physical activity), as well as encouraging information, “take home messages” at the end of each theme, and reminders to use the self-monitoring features. The self-monitoring features provided the possibility to track weight gain, diet, and physical activity and to set a physical activity goal for MVPA (minutes per week). Participants received predesigned feedback based on their registrations of diet and physical activity in accordance with national guidelines [25,26]. Feedback consisted of a graphical visualization of the registrations where participants could review their reported diet, physical activity, and weight gain over time in relation to the recommendations. Participants also received feedback in text and a “traffic light” (ie, green: reached the recommendation; yellow: close to reaching the recommendation; red: far from reaching the recommendation) indicating compliance with recommendations following registration of diet and physical activity. Furthermore, the weight gain chart showed their individual GWG in relation to the recommended weight gain (according to the recommendations provided by the National Academy of Medicine [31], calculated from their self-reported pre-pregnancy BMI). Participants allocated to the intervention group were informed of the features in the app, how to download it (from AppStore [iOS] or Google Play [Android]), and were instructed to use the app as much as they preferred. Participants were registered as app users by the researchers, whereby they received an SMS text message with a link to a downloading site where they could download the app (free of charge). At follow-up, participants filled in a questionnaire regarding their satisfaction and usage of the app.

### Sample Size, Randomization, and Blinding

We estimated that 226 women (113 in each group) would provide 80% power (α=.05, 2 sided), assuming a common SD in GWG of 4 kg [32] to detect a difference of 1.5 kg between the control and intervention group. Considering a maximal loss to follow-up of 25% we recruited just over 300 women. After completion of baseline measures participants were randomized in a 1:1 ratio to either the control or intervention group using restricted randomization generated using STATA (version 13; StataCorp). Allocation concealment was ensured by using opaque envelopes (PH) which was opened by the assessor after completion of all the baseline measures whereby the participant was informed of their allocation (ie, the intervention or control group). The participants and outcome assessors were not blinded to the allocation due to the nature of the intervention.

### Outcomes

The primary outcome was GWG between baseline (gestational week 14) and the follow-up measurement (gestational week 37). Secondary outcomes included body fatness, dietary habits (Swedish Healthy Eating Index), physical activity (time spent in MVPA), glycemia, and insulin resistance. All outcomes were assessed in gestational weeks 14 and 37.

#### Gestational Weight Gain

Body weight was measured after an overnight fast when the participant was wearing underwear using standardized procedures (Bod Pod; COSMED). Subsequently, GWG was calculated as the difference in body weight (in kg) between the baseline measurement (gestational week 14) and the follow-up measurement (gestational week 37). Furthermore, GWG between gestational weeks 14 and 37 was expressed per week (kg/week). Subsequently, we applied the pre-pregnancy BMI-specific GWG recommendations for the second and third trimester proposed by the National Academy of Medicine to categorize GWG for each woman as inadequate, adequate, and excessive (ie, underweight: 0.44-0.58 kg/week; normal weight: 0.35-0.50 kg/week; overweight: 0.23-0.33 kg/week; and obesity: 0.17-0.27 kg/week) [31].

#### Body Fatness

Body composition was measured using Bod Pod (COSMED) with accompanying software version 5.2.0 as described previously [33]. In short, the Bod Pod measures body volume using air-displacement plethysmography and body density was then derived by dividing body weight with body volume. Fat and fat-free mass were calculated using densities specified for the gestational age at baseline and follow-up measurements [34].

#### Dietary Habits

The web-based dietary recall method Riksmaten FLEX developed by the Swedish National Food Agency [35] adapted to pregnant women was used to assess dietary habits. The method utilized a repeated 24-hour recall approach over 3 days [35]. Upon the first log-in, participants were instructed to register their dietary intake for that day and the day before. The third day was automatically assigned to occur within 7 days of the first registration, on either a weekday or weekend day depending on what day the first day of registration was to ensure that registrations included both weekdays and weekend days [35].

The design of Riksmaten FLEX has been described in detail elsewhere [35]. In short, it consists of food items and prespecified dishes that participants can choose from. Once the correct food item or dish has been selected, participants defined portion sizes by choosing among pictures demonstrating various amounts of foods. Registrations were then linked to the Swedish National Food Composition Database, providing intakes of energy, macronutrients, and micronutrients [35]. In accordance with Moraeus et al [35], registered days with ≥3500 kcal or days ≤800 kcal were checked in detail by the research group to detect inaccurate energy intakes. In total, intakes for 1 (at baseline) and 3 days (at follow-up) were considered implausible and excluded. Intakes of selected food groups (ie, fruits, vegetables, red meat, fish, and shellfish) and macronutrients were summarized and averaged for each participant and day.

To assess diet quality, we calculated the Swedish Healthy Eating Index score [36], based on the Nordic Nutrition Recommendations [25], for each woman. The score consists of 9 components and was calculated based on intakes of fruit and vegetables (g/day), fish and shellfish (g/day), red meat (g/week), fiber (g/MJ), wholegrain (g/MJ), polyunsaturated fat (E%), monounsaturated fat (E%), saturated fat (E%), and sucrose (E%) as described elsewhere [36]. The score for each item ranged from 0 to 1 and the total score ranged from 0 to 9, with a higher score indicating better compliance with dietary guidelines [36].

#### Physical Activity

An ActiGraph wGT3x-BT (ActiGraph) accelerometer was used to assess physical activity. Participants were instructed to wear the accelerometer on the wrist for 7 consecutive 24-hour periods and to only remove it when engaging in water activities. The accelerometer was programmed to register accelerations at 100 Hz and participants filled in a diary where they reported sleep time and nonwear time which were used in the analysis to confirm sleep time. Participant data with at least one valid day were included. A valid day was defined as one-third or more of the 24-hour period being wear time, two-thirds or more of the wake time being wear time, and two-thirds or more of the sleep time being wear time. Participants who were not able to wear the accelerometer on the wrist (eg, health care workers due to hygiene restrictions at the workplace) were instructed to wear it on the hip instead, both at baseline and at follow-up (baseline n=23; follow-up n=18). Appropriate thresholds to identify MVPA were used for wrist- (ie, 100 m*g*) and hip-worn accelerometers (ie, 70 m*g*) [37]. Daily average MVPA was calculated as the weighted mean of weekdays and weekend days, that is, {([mean of weekdays × 5] + [mean of weekend days × 2])/7}. The intervention effect was very comparable when excluding the 29 women that wore the accelerometer on the hip or had less than 4 valid days [38] of recorded physical activity at both baseline and follow-up (results not shown). Data processing was conducted using the software program R [39] and the package GGIR [40].

#### Glycemia and Insulin Resistance

Blood samples were drawn after an overnight fast. Concentrations of glucose and insulin were analyzed on a Cobas 602 (Roche Diagnostics Scandinavia AB) at the Department of Clinical Chemistry, Linköping University Hospital. Insulin resistance was assessed by using the Homeostatic Model Assessment for Insulin Resistance (HOMA-IR) according to Matthews et al [41] and was calculated as fasting insulin [µU/L] × fasting glucose [mmol/L])/22.5.

### Statistical Analysis

#### Effectiveness of the Intervention

All statistical analyses were conducted in accordance with the study protocol [23] and the CONSORT-EHEALTH statement [24]. Analyses followed principles of intention-to-treat and were performed in R version 3.6.3 (R Foundation for Statistical Computing). Null hypotheses were tested at the .05 significance level (2 sided). Missing data were imputed by means of multiple imputations in which the value of missing observations is predicted using available data. We used multiple imputations with chained equations [42] employing the analysis regression model as the prediction model within iterations. A total of 500 data sets were imputed for each analysis (predictive mean matching with 50 iterations) and analyses were pooled using Rubin’s rules [43]. We also conducted complete case analyses for all outcomes. As described in the study protocol [23], we planned for per-protocol analysis including women who had used the app at least once and who had data on GWG at follow-up; however, only 1 participant was removed in the per-protocol analyses compared with the complete case analyses, and findings were unchanged. Therefore, we only report multiple imputations analyses and complete case analyses.

To contrast differences in primary (GWG in kg) and secondary outcomes (Swedish Healthy Eating Index, MVPA, body fatness, glycemia, and insulin resistance) between the 2 groups (intervention vs control) we estimated linear regression models. More specifically, for the primary outcome GWG, we regressed follow-up weight in gestational week 37 on group allocation and adjusted for baseline weight in gestational week 14 (crude model). This procedure has the advantage of being robust to imbalances at baseline and regression toward the mean [44]. The group coefficient in this model provides an estimate of the expected difference in GWG between 2 participants with the same baseline weight who have received different treatment (intervention vs control). Thereafter a second regression model was fitted with additional adjustments for pre-pregnancy BMI (underweight and normal weight vs overweight and obesity), parity (0 vs ≥1), and educational attainment (university degree vs no university degree) (adjusted model). Corresponding models were fitted for all secondary outcomes (Swedish Healthy Eating Index, physical activity, body fatness, glycemia, and insulin resistance). As planned, we also estimated effect modifications of the intervention on the primary outcome (GWG), by extending the regression model with interactions between group allocation and pre-pregnancy BMI, parity, and educational attainment, respectively.

#### Sensitivity and Complementary Analyses

We conducted the following sensitivity and complementary analyses. First, in accordance with our protocol [23], we conducted a sensitivity analysis excluding women diagnosed with gestational diabetes or pre-eclampsia (n=7) before the follow-up measurement and the intervention effect was comparable (results not shown). Second, as in one of our recently published mHealth trials [45], we extended our prespecified statistical analysis plan with a Bayesian analysis for the primary outcome. Thus, we analyzed the interaction effect between group allocation and pre-pregnancy BMI using a Bayesian analysis [46]. This was done as the trial was not planned to be powered to detect this effect, and the Bayesian analysis allowed us to calculate the posterior probability of an interaction effect despite the null hypothesis not being rejected [47,48]. For the Bayesian analyses, imputation was done within the model estimation, that is, within the Markov Chain Monte Carlo simulations. For each iteration, missing data were replaced with draws from a normal distribution specified by the sample parameters of the regression model being estimated.

## Results

### Participants

**Figure 2:**
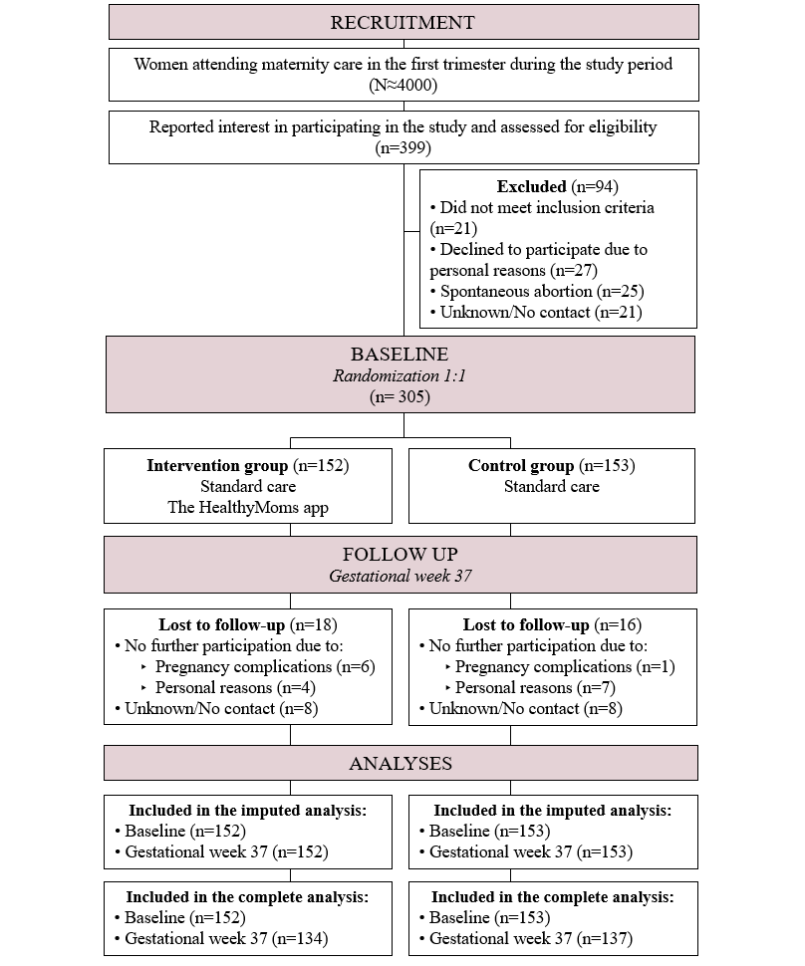
Flowchart of the HealthyMoms trial.

As shown in Figure 2, approximately 4000 eligible pregnant women entered maternity health care during the recruitment period (October 2017 to March 2020), and 399 women expressed interest to the research group to participate in the study. Ninety-four women were excluded because (1) they did not meet all the inclusion criteria (n=21), (2) declined participation after full information (n=27), (3) had a miscarriage before enrollment (n=25), or (4) did not reply after expressing interest to participate (n=21). A total of 305 pregnant women were enrolled and randomized and their baseline characteristics are provided in Table 1. At baseline, their mean age was 31 (SD 4) years; 57.4% (175/305) were nulliparous; and 2.0% (6/305) were underweight, 69.5% (212/305) normal weight, and 28.5% (87/305) had overweight or obesity pre-pregnancy. As shown in Table 1, there were no major differences in baseline characteristics between the women in the intervention and control group. In total, 152 participants were allocated to the intervention group, of which 151 downloaded the app, and all participants were included in the analyses regardless of app usage. Table 2 reports the self-reported satisfaction of the HealthyMoms app. For instance, 77.6% (104/134) fully or largely agreed with the statement that they were satisfied with the app and another 13.4% (18/134) agreed to some extent. Additionally, the majority of participants (82.8%, 111/134) reported using the app at least once per week and only a few (17.2%, 23/134) reported using the app 2 to 3 times per month or less. Additional subjective data on app usage are provided in Multimedia Appendix 2. Furthermore, objective measures showed the following mean usage of the registration features: physical activity: 1.6 (SD 2.1) times/week; self-monitoring for weight: 0.7 (SD 0.8) times/week; and diet registration 0.2 (SD 0.3) times/week.

**Table 1:** Baseline characteristics of the women in the HealthyMoms trial.

Characteristics			All women (n=305)	Intervention (n=152)	Control (n=153)
**Pre-pregnancy characteristics, n (%)**					
	**Parity^a^**				
		0	175 (57.4)	86 (56.6)	89 (58.2)
		≥1	130 (42.6)	66 (43.4)	64 (41.8)
	**Educational attainment^a^**				
		Primary school (9 years)	2 (0.7)	0 (0.0)	2 (1.3)
		High school (12 years)	66 (21.6)	37 (24.3)	29 (19.0)
		University degree	237 (77.7)	115 (75.7)	122 (79.7)
	**Pre-pregnancy BMI^a,b^**				
		Underweight (<18.5 kg/m^2^)	6 (2.0)	1 (0.7)	5 (3.3)
		Normal weight (18.5-24.9 kg/m^2^)	212 (69.5)	103 (67.8)	109 (71.2)
		Overweight (25.0-29.9 kg/m^2^)	67 (22.0)	34 (22.4)	33 (21.6)
		Obesity (≥30.0 kg/m^2^)	20 (6.6)	14 (9.2)	6 (3.9)
**Measured variables in gestational week 14 (baseline), mean (SD)**					
	**General characteristics^a^**				
		Gestational week	13.9 (0.7)	13.8 (0.6)	14.0 (0.7)
		Age (years)	31.3 (4.1)	31.4 (4.3)	31.3 (3.8)
	**Anthropometry^a^**				
		Weight (kg)	67.7 (11.5)	68.3 (12.8)	67.0 (10.2)
		Height (m)	1.67 (0.06)	1.66 (0.06)	1.68 (0.06)
		BMI (kg/m^2^)	24.2 (3.8)	24.7 (4.3)	23.8 (3.2)
		Fat mass index (kg/m^2^)	8.0 (3.2)	8.4 (3.6)	7.6 (2.6)
		Fat free mass index (kg/m^2^)	16.2 (1.3)	16.2 (1.4)	16.2 (1.3)
	Swedish Healthy Eating Index Score^c^		6.66 (0.98)	6.54 (0.98)	6.79 (0.97)
	Moderate-to-vigorous physical activity (min/day)^d^		39.2 (24.0)	38.7 (24.6)	39.8 (23.5)
	Glycemia (mmol/L)^e^		4.8 (0.3)	4.8 (0.3)	4.8 (0.3)
	Homeostatic Model Assessment for Insulin Resistance^e^		1.4 (0.7)	1.4 (0.8)	1.4 (0.7)

^a^All women (n=305): intervention group (n=152) + control group (n=153).

^b^Based on self-reported pre-pregnancy weight and height.

^c^All women (n=302): intervention group (n=151) + control group (n=151).

^d^All women (n=296); intervention group (n=146) + control group (n=150).

^e^All women (n=304); intervention group (n=151) + control group (n=153).

**Table 2:** Self-reported app satisfaction in the intervention group (n=134) at the follow-up measurement. Participants responded to the following statements with the 6 alternatives shown.

Statement	Strongly disagree, n (%)	Agree to a small extent, n (%)	Agree to some extent, n (%)	Strongly agree, n (%)	Fully agree, n (%)	Do not know, n (%)
I am satisfied with the app	2 (1.5)	5 (3.7)	18 (13.4)	66 (49.3)	38 (28.4)	5 (3.7)
The app has been a good support for a healthy weight gain during pregnancy	9 (6.7)	18 (13.4)	39 (29.1)	32 (23.9)	20 (14.9)	16 (11.9)
The app has been a good support for healthy food habits	12 (9.0)	16 (11.9)	42 (31.3)	41 (30.6)	11 (8.2)	12 (9.0)
The app has been a good support for exercise habits	15 (11.2)	16 (11.9)	29 (21.6)	44 (32.8)	20 (14.9)	10 (7.5)
The app has given me insight regarding my food habits	26 (19.4)	16 (11.9)	39 (29.1)	31 (23.1)	9 (6.7)	13 (9.7)
The app has given me insight regarding how physically active I am	28 (20.9)	16 (11.9)	32 (23.9)	36 (26.9)	13 (9.7)	9 (6.7)
I think that the HealthyMoms app is better than other similar apps	3 (2.2)	10 (7.5)	31 (23.1)	24 (17.9)	9 (6.7)	57 (42.5)
I would recommend other pregnant women to use the HealthyMoms app	3 (2.2)	7 (5.2)	16 (11.9)	45 (33.6)	57 (42.5)	6 (4.5)

### Effectiveness of the Intervention (Primary Outcomes)

Table 3 presents the intervention effects for the primary outcome (GWG) for the crude model as well as the model adjusted for pre-pregnancy BMI, parity, and educational attainment for the imputed (n=305) and the complete cases analyses (n=271), respectively. Overall, results showed no statistically significant difference between the groups on GWG (–0.20 kg; 95% CI –0.98 to 0.59; *P*=.62 for the intervention group vs the control, n=305). Furthermore, as shown in Table 3, results were similar when taking baseline weight, pre-pregnancy BMI, parity, and educational attainment into account as well as in analyses with complete cases. Overall, 13.3% (36/271), 36.9% (100/271), and 49.8% (135/271) of the women gained below, within, and above the recommendations by the National Academy of Medicine, and there was no statistical difference in adherence to the recommendations between the intervention and control group (Table 4).

**Table 3:** Intervention effect on gestational weight gain (primary outcome) assessed using regression analysis^a,b,c^.

Model	Imputed data analysis (n=305)		Complete cases analysis (n=271)	
	Coefficient (95% CI)	*P* value	Coefficient (95% CI)	*P* value
Crude	–0.20 (–0.98 to 0.59)	.62	–0.22 (–1.00 to 0.56)	.58
Adjusted	–0.20 (–1.00 to 0.60)	.62	–0.24 (–1.01 to 0.54)	.55

^a^Regression analysis of follow-up measure of weight on group allocation. The coefficient is interpreted as the estimated effect of the intervention compared with the control adjusted for baseline weight (crude model), BMI category (underweight and normal weight vs overweight and obesity), parity (0 vs 1 or more), and educational attainment (university degree vs no university degree) (adjusted model).

^b^Baseline, n=305 (152 intervention and 153 control); Follow-up, n=271 (134 intervention and 137 control).

^c^At baseline, the mean bodyweight (kg) for the intervention and control group was 68.3 (SD 12.8) and 67.0 (SD 10.2), respectively, whereas at follow-up the corresponding values were 78.7 (SD 13.1) and 77.3 (SD 10.6).

**Table 4:** Intervention effect on gestational weight gain according to National Academy of Medicine’s recommendations.

Outcome	Descriptive data, n (%)		Intervention effect using regression analysis^a^			
	Group		Imputed data analysis (n=305)		Complete cases analysis (n=271)	
	Intervention (n=134)	Control (n=137)	Odds ratio^a^ (95% CI)	*P* value	Odds ratio^a^ (95% CI)	*P* value
Excessive GWG^b,c^	67 (50.0)	68 (49.6)	0.75 (0.43-1.32)	.31	0.75 (0.43-1.32)	.32
Adequate GWG^b^	52 (38.8)	48 (35.0)	Reference		Reference	
Inadequate GWG^b^	15 (11.2)	21 (15.3)	0.66 (0.30-1.43)	.29	0.66 (0.30-1.44)	.29

^a^Regression analysis of gestational weight gain on group allocation. The coefficient is interpreted as the estimated effect of the intervention compared with the control adjusted for baseline body weight, BMI category (underweight and normal weight vs overweight and obesity), parity (0 vs 1 or more), and educational attainment (university degree vs no university degree).

^b^GWG was calculated as the difference between weight at follow-up and baseline, which then was divided by gestational weeks to obtain GWG expressed as kg/week. This GWG (kg/week) was compared to the weekly GWG recommendations by the National Academy of Medicine for the second and third trimesters to classify GWG as excessive, adequate, or inadequate.

^c^GWG: gestational weight gain.

There was no statistically significant interaction effect for parity or educational attainment (results not shown); however, data indicated that there was a marked interaction between pre-pregnancy BMI and group allocation with the intervention being more effective in women with overweight and obesity compared with those who were underweight and normal weight. Thus, for women with overweight and obesity, GWG in the intervention group was 1.33 kg (95% CI –2.92 to 0.26, *P*=.10, n=305) lower than those in the control group when also accounting for parity and educational attainment. In the complete case analysis, the interaction effect was stronger and statistically significant (–1.67 kg; 95% CI –3.26 to –0.09; *P*=.031, n=271).

**Figure 3:**
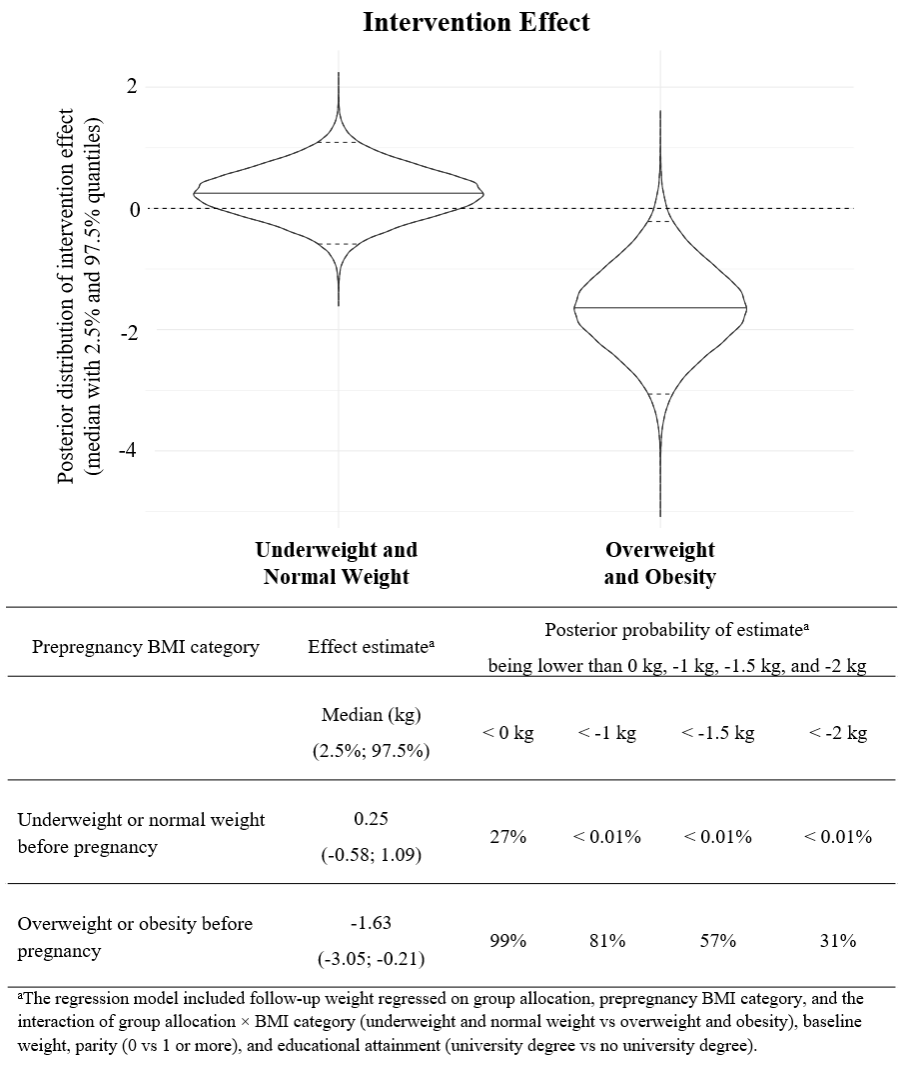
Bayesian analysis (with imputation, n=305) of the intervention effect on gestational weight gain according to prepregnancy BMI.

The interaction effect was furthermore supported by the results from a Bayesian estimation of the same interaction model (Figure 3). The probability that the expected GWG in the intervention group was less than that in the control group (ie, the intervention had any effect on GWG) was only 27% among underweight and normal weight women; however, among women with overweight and obesity it was 99%. Furthermore, the probability that this effect was over 1 and 1.5 kg among women with overweight and obesity was 81% and 57%, respectively.

### Secondary Outcomes

Table 5 shows the corresponding results for the secondary outcomes. The intervention group had higher scores for the Swedish Healthy Eating Index at follow-up than the control group (0.27; 95% CI 0.05-0.50; *P*=.017) when adjusting for baseline values as well as for pre-pregnancy BMI, parity, and educational attainment. The difference in the Swedish Healthy Eating Index was driven by an overall shift, with slightly higher scores in 7 out of 9 components, toward a healthier diet as indicated by higher intakes of fruits and vegetables and with a statistically significant observed reduction in the intake of red meat (*P*=.027) (Multimedia Appendix 3). No statistically significant differences in MVPA or any of the other secondary outcomes at gestational week 37 were observed between the intervention and control group (*P*≥.21).

**Table 5:** Intervention effect on the secondary outcomes.

Outcome		Descriptive data, mean (SD)		Intervention effect using regression analysis^a^			
		Group		Imputed data analysis		Complete cases analysis	
		Intervention	Control	Coefficient^a^ (95% CI)	*P* value	Coefficient^a^ (95% CI)	*P* value
**Swedish Healthy Eating index (points)**							
	Baseline (n=302)^b^	6.54 (0.98)	6.79 (0.97)	0.27 (0.05 to 0.50)	.017	0.27 (0.05 to 0.50)	.018
	Follow-up (n=269)^b^	6.53 (0.94)	6.38 (1.07)				
**Moderate-to-vigorous physical activity (min/day)**							
	Baseline (n=296)^c^	38.7 (24.6)	39.8 (23.5)	–0.76 (–5.34 to 3.80)	.74	–1.01 (–5.66 to 3.62)	.67
	Follow-up (n=267)^c^	26.3 (19.0)	27.8 (24.7)				
**Fat mass (kg)**							
	Baseline (n=305)^d^	23.4 (10.1)	21.5 (7.6)	0.05 (–0.65 to 0.76)	.88	–0.03 (–0.71 to 0.64)	.92
	Follow-up (n=268)^d^	26.8 (9.7)	24.7 (7.5)				
**Fat-free mass (kg)**							
	Baseline (n=305)^d^	45.0 (4.8)	45.6 (4.8)	–0.09 (–0.46 to 0.28)	.64	–0.07 (–0.45 to 0.30)	.70
	Follow-up (n=268)^d^	51.9 (5.4)	52.5 (5.4)				
**Glycemia (mmol/L)**							
	Baseline (n=304)^e^	4.8 (0.3)	4.8 (0.3)	0.06 (–0.03 to 0.15)	.21	0.06 (–0.03 to 0.14)	.18
	Follow-up (n=263)^e^	4.7 (0.5)	4.6 (0.3)				
**Homeostatic Model Assessment for Insulin Resistance**							
	Baseline (n=304)^e^	1.41 (0.76)	1.36 (0.65)	0.10 (–0.13 to 0.34)	.39	0.12 (–0.11 to 0.36)	.31
	Follow-up (n=263)^e^	2.41 (1.36)	2.19 (0.98)				

^a^Regression analysis of follow-up measure of secondary outcome on group allocation. The coefficient is interpreted as the estimated effect of the intervention compared with the control adjusted for baseline value of the secondary outcome, BMI category (underweight and normal weight vs overweight and obesity), parity (0 vs 1 or more), and educational attainment (university degree vs no university degree). Imputed data analysis included data for all 305 women and the complete cases analysis included data for 263-269 women.

^b^Baseline, n=302 (151 intervention and 151 control); follow-up, n=269 (135 intervention and 134 control).

^c^Baseline, n=296 (146 intervention and 150 control); follow-up, n=267 (132 intervention and 135 control). Number of valid days for accelerometry: intervention group (baseline: 6.5 [SD 1.1] days; follow-up: 6.7 [SD 0.8] days); control group (baseline: 6.7 [SD 0.9] days; follow-up: 6.7 [SD 1.1] days). Average wear time for valid days: intervention group (baseline: 99.0%; follow-up: 97.5%); control group (baseline: 98.7%; follow-up: 98.4%).

^d^Baseline, n=305 (152 intervention and 153 control); follow-up, n=268 (133 intervention and 135 control).

^e^Baseline, n=304 (151 intervention and 153 control); follow-up, n=263 (130 intervention and 133 control).

## Discussion

### Principal Findings

This study is the first to examine the effectiveness of a comprehensive intervention delivered solely via an app on GWG, body fatness, dietary habits, physical activity, glycemia, and insulin resistance in pregnant women from all BMI categories. We did not observe any statistically significant effect on GWG; however, there was some evidence that women with overweight and obesity before pregnancy gained less weight in the intervention group as compared with the control group in the imputed analyses (–1.33 kg; 95% CI –2.92 to 0.26; *P*=.10) and the completers-only analyses (–1.67 kg; 95% CI –3.26 to –0.09; *P*=.031). Furthermore, a Bayesian analysis supported that the intervention was more effective among women with overweight and obesity. Regarding secondary outcomes, we found no effect on body fatness, MVPA, glycemia, and insulin resistance; however, participants in the intervention group had a higher score in the Swedish Healthy Eating Index in gestational week 37 compared with the control group.

### Comparison With Previous Studies

Previous studies of apps promoting healthy GWG have been pilot studies [15] and have only included women with overweight and obesity. Nevertheless, our study can be compared with the 3-arm randomized controlled trial by Olson et al [19] in pregnant women in the United States (n=1689, BMI ≥18.5-35 kg/m^2^) evaluating the effectiveness of access to a website aimed to support healthy weight gain, diet, and physical activity in pregnancy. Similar to Olson et al [19], we found no statistically significant effect on total GWG and the participants were primarily normal weight preconception. Interestingly, as stated above, we observed an interaction between pre-pregnancy BMI and group allocation on GWG, suggesting that women with overweight and obesity benefitted more from the intervention. These results were further complemented by our Bayesian analysis showing a 99% probability that the intervention had any effect and an 81% probability that this effect was larger than 1 kg. Our results are also in line with a previous pilot study of an mHealth intervention among pregnant women with overweight and obesity [18], where participants in the intervention group gained less weight than those in the control group (7.8 kg [SD 4.7] kg vs 9.7 kg [SD 3.9]; *P*=.041). Additionally, the intervention effect estimated in our study using only an app is comparable to previous interventions in pregnant women with overweight and obesity relying on traditional face-to-face counseling [10]. This suggests that mHealth interventions may be as effective as these traditional approaches, while using less resources, being more cost-effective, and having greater reachability. Taken together, mHealth interventions have potential to be made readily available to many women at the touch of a button.

Another main finding is that we observed a statistically significant higher Swedish Healthy Eating Index score in the intervention group compared with the control group. As shown in Multimedia Appendix 3, from the individual components of the score, the higher score was driven by an overall shift toward a healthier diet, including a statistically significant reduction in the intake of red meat. The effect on red meat may be explained by the fact that the HealthyMoms app was carefully designed to also highlight the benefits of plant-based diets by providing information and weekly menus with only vegetarian foods (in addition to the mixed diet menus) in the recipe module. Comparisons with previous mHealth studies aiming to improve dietary behaviors are difficult because dietary interventions are complex with various focus and intervention features. Additionally, studies have used different outcome measures (eg, food frequency questionnaires that may be less sensitive to capture variations in dietary intake [18]). Nevertheless, it is interesting to note that similar to our study, Ainscough et al [22] as well as Li et al [49] observed statistically significant effects on dietary outcomes using digital interventions in pregnant women with overweight and obesity. In summary, our results provide evidence that a 6-month behavioral intervention through an app could potentially improve dietary behaviors in pregnant women and thus represent a key target for future mHealth GWG interventions.

In contrast to the positive findings for dietary behaviors, we observed no effect on MVPA in this study. A few pilot mHealth studies in pregnant women have shown a beneficial effect on physical activity [18,50], while others have not [51]; however, these studies evaluated self-reported outcomes [18] or utilized steps from Fitbit manually imputed by participants [51] or had a short duration [50]. Noteworthy, our results showing an effect on diet but not on MVPA may be reconciled with previous face-to-face interventions which have shown stronger effects on GWG for interventions targeting dietary behaviors compared with physical activity [52]. It is possible that the relatively modest effect on diet coupled with the lack of an effect on physical activity may explain why the intervention did not have any overall effect on GWG. One potential explanation for not observing an effect on MVPA could be that the third trimester is characterized by an increase in body mass and fetal growth which may inhibit the capacity to perform certain exercises, as well as symptoms such as pelvic pain that may decrease the ability or the motivation to be physically active. Thus, at this stage of gestation, reductions in physical activity are common [53] and it may be difficult to detect if the intervention has counteracted a natural decline. In this context it is also relevant to note that Choi et al [51] reported that it was difficult to recruit inactive women that wanted to increase physical activity during pregnancy for an intervention. Consequently, further studies to explore the potential of mHealth interventions to improve physical activity behaviors during pregnancy are required and preferably such studies should include objective measures of physical activity at multiple timepoints from early pregnancy.

### Strengths and Limitations

The HealthyMoms trial has several strengths. These include the randomized control design, high compliance (88.8% completion rate, 271/305) which provided adequate power to assess our outcomes, and the use of accurate and objective methods to assess primary and secondary outcomes (ie, measurements of body weight and body fatness using Bod Pod and objective measures of physical activity). Furthermore, another strength is that the intervention is theory-informed and uses key behavioral change techniques [27,28] which have been shown to be important elements with regard to intervention effectiveness and engagement in pregnant women [15]. More specifically, we utilized goal setting, self-monitoring, and feedback which have been demonstrated to increase effectiveness of digital dietary and physical activity interventions targeting pregnant women [15]. An additional strength of the study is the inclusion of women from all BMI categories and not only women with overweight and obesity. This study has also limitations to be acknowledged. Our study sample contained a larger proportion of women with a university degree compared with women in the general population (78% vs approximately 47%) [54]. This may influence generalizability to women with lower socioeconomic status although we found no evidence that the intervention effect differed according to educational attainment. Correspondingly, the prevalence of women with overweight and obesity in this study was somewhat lower than the general pregnant population in Sweden (29% vs 37%) [4]. Nevertheless, the prevalence of inadequate and excessive GWG in the trial (13% inadequate, 50% excessive) was similar to the general population (18% inadequate, 47% excessive) [4]. Besides, because we did not conduct randomization stratified by pre-pregnancy BMI, the number of women with overweight/obesity was slightly different in the intervention and control group which may somewhat decrease statistical power. Nevertheless, we were able to observe effects on GWG in women with overweight and obesity, although future studies could include a larger sample of such women. Furthermore, an inherent limitation is the risk of recall bias and social desirability in dietary assessments since established methods rely on self-report. However, we utilized a comprehensive and feasible web-based 24-hour recall method, which has previously been used in a national survey in Sweden [35]. Compared with other commonly used methods, 24-hour recalls are to a lower extent associated with misreporting of dietary intakes [55]. Although we mainly utilized objective study measures, another limitation is that the assessors were only masked to the randomization assignment for the baseline measures. Furthermore, it cannot be excluded that the intervention effect was diluted as the control group was carefully measured in terms of body composition and weight, diet, and physical activity, in order to compare the groups regarding outcome measures, which may have influenced their behavior. Finally, another limitation of the study is that the intervention is only available in Swedish and thus women not proficient in the Swedish language were excluded. Thus, topics for future research should include pregnant women from various socioeconomic and migrant backgrounds [56].

### Clinical and Public Health Relevance

The HealthyMoms trial provides several important findings. Although we did not observe a statistically significant overall effect on the primary outcome (GWG), our findings indicate a meaningful effect in pregnant women with overweight or obesity compared with standard care, with a similar effect as seen in traditional face-to-face interventions [10]. Furthermore, we observed a positive overall effect of the intervention on dietary habits, regardless of pre-pregnancy BMI. Self-reported data on app usage indicated high engagement and satisfaction by participants, which was also confirmed in a detailed qualitative [57] and a quantitative evaluation in a subsample (to be reported elsewhere). Altogether, these results indicate that the HealthyMoms app has potential to be a valuable tool to promote healthy diet and weight gain in pregnant women. Access to scalable and cost-effective interventions such as the HealthyMoms app may be of importance in order to counteract the high prevalence of excessive GWG in Sweden and globally [1,2,4] and its negative implications on pregnancy complications and long-term maternal and fetal health [2,5]. More specifically, the HealthyMoms app is particularly attractive in this context because it is a fully automated stand-alone intervention, which does not rely on additional resources from health care. The next step is to estimate the effectiveness of the HealthyMoms app across Sweden including women from various socioeconomic and migrant backgrounds as well as additional pregnancy outcomes such as pre-eclampsia and gestational diabetes. If clinical variables collected routinely in health care are used as outcomes, access to the app could be given already in the first trimester which may increase the effectiveness of the HealthyMoms app. Altogether, such an evaluation will be important considering the great lack of large-scale digital interventions in this field.

### Conclusions

Although we found no overall effect on GWG, our results demonstrate the potential of a smartphone app (HealthyMoms) to promote healthy dietary behaviors as well as to decrease weight gain during pregnancy in women with overweight and obesity, and with a similar effect as in traditional interventions. Thus, this intervention, solely delivered through an app, has potential to be useful for promoting a healthy lifestyle during pregnancy in many women while using less resources from health care.

## Appendix

Multimedia Appendix 1 CONSORT eHealth checklist.

Multimedia Appendix 2 Additional self-reported data on app usage and satisfaction in the intervention group (n=134) at the follow-up measurement. Participants responded to the following statements with the 6 alternatives shown.

Multimedia Appendix 3 Intervention effect on the components in the Swedish Healthy Eating Index.

## Abbreviations

GWG: gestational weight gain
HOMA-IR: Homeostatic Model Assessment for Insulin Resistance
mHealth: mobile health
MVPA: moderate-to-vigorous physical activity

## References

[1] Goldstein RF, Abell SK, Ranasinha S, Misso ML, Boyle JA, Harrison CL, Black MH, Li N, Hu G, Corrado F, Hegaard H, Kim YJ, Haugen M, Song WO, Kim MH, Bogaerts A, Devlieger R, Chung JH, Teede HJ (2018). Gestational weight gain across continents and ethnicity: systematic review and meta-analysis of maternal and infant outcomes in more than one million women. BMC Med.

[2] Goldstein RF, Abell SK, Ranasinha S, Misso M, Boyle JA, Black MH, Li N, Hu G, Corrado F, Rode L, Kim YJ, Haugen M, Song WO, Kim MH, Bogaerts A, Devlieger R, Chung JH, Teede HJ (2017). Association of Gestational Weight Gain With Maternal and Infant Outcomes: A Systematic Review and Meta-analysis. JAMA.

[3] Henriksson P, Eriksson B, Forsum E, Löf M (2015). Gestational weight gain according to Institute of Medicine recommendations in relation to infant size and body composition. Pediatr Obes.

[4] Henriksson P, Sandborg J, Blomberg M, Nowicka P, Petersson K, Bendtsen M, Rosell M, Löf M (2020). Body mass index and gestational weight gain in migrant women by birth regions compared with Swedish-born women: A registry linkage study of 0.5 million pregnancies. PLoS One.

[5] Voerman E, Santos S, Inskip H, Amiano P, Barros H, Charles M, Chatzi L, Chrousos GP, Corpeleijn E, Crozier S, Doyon M, Eggesbø M, Fantini MP, Farchi S, Forastiere F, Georgiu V, Gori D, Hanke W, Hertz-Picciotto I, Heude B, Hivert M, Hryhorczuk D, Iñiguez C, Karvonen AM, Küpers LK, Lagström H, Lawlor DA, Lehmann I, Magnus P, Majewska R, Mäkelä J, Manios Y, Mommers M, Morgen CS, Moschonis G, Nohr EA, Nybo Andersen A, Oken E, Pac A, Papadopoulou E, Pekkanen J, Pizzi C, Polanska K, Porta D, Richiardi L, Rifas-Shiman SL, Roeleveld N, Ronfani L, Santos AC, Standl M, Stigum H, Stoltenberg C, Thiering E, Thijs C, Torrent M, Trnovec T, van Gelder MMHJ, van Rossem L, von Berg A, Vrijheid M, Wijga A, Zvinchuk O, Sørensen TIA, Godfrey K, Jaddoe VWV, Gaillard R, LifeCycle Project-Maternal ObesityChildhood Outcomes Study Group (2019). Association of Gestational Weight Gain With Adverse Maternal and Infant Outcomes. JAMA.

[6] Han Z, Lutsiv O, Mulla S, Rosen A, Beyene J, McDonald SD, Knowledge Synthesis Group (2011). Low gestational weight gain and the risk of preterm birth and low birthweight: a systematic review and meta-analyses. Acta Obstet Gynecol Scand.

[7] International Weight Management in Pregnancy (i-WIP) Collaborative Group (2017). Effect of diet and physical activity based interventions in pregnancy on gestational weight gain and pregnancy outcomes: meta-analysis of individual participant data from randomised trials. BMJ.

[8] Peaceman AM, Clifton RG, Phelan S, Gallagher D, Evans M, Redman LM, Knowler WC, Joshipura K, Haire-Joshu D, Yanovski SZ, Couch KA, Drews KL, Franks PW, Klein S, Martin CK, Pi-Sunyer X, Thom EA, Van Horn L, Wing RR, Cahill AG, LIFE‐Moms Research Group (2018). Lifestyle Interventions Limit Gestational Weight Gain in Women with Overweight or Obesity: LIFE-Moms Prospective Meta-Analysis. Obesity (Silver Spring).

[9] Muktabhant B, Lawrie TA, Lumbiganon P, Laopaiboon M (2015). Diet or exercise, or both, for preventing excessive weight gain in pregnancy. Cochrane Database Syst Rev.

[10] Shieh C, Cullen DL, Pike C, Pressler SJ (2018). Intervention strategies for preventing excessive gestational weight gain: systematic review and meta-analysis. Obes Rev.

[11] Lau Y, Klainin-Yobas P, Htun TP, Wong SN, Tan KL, Ho-Lim ST, Chi C, Tsai C, Ong KW, Shorey S, Tam WSW (2017). Electronic-based lifestyle interventions in overweight or obese perinatal women: a systematic review and meta-analysis. Obes Rev.

[12] Mateo GF, Granado-Font E, Ferré-Grau C, Montaña-Carreras X (2015). Mobile Phone Apps to Promote Weight Loss and Increase Physical Activity: A Systematic Review and Meta-Analysis. J Med Internet Res.

[13] Schoeppe S, Alley S, Lippevelde WV, Bray NA, Williams SL, Duncan MJ, Vandelanotte C (2016). Efficacy of interventions that use apps to improve diet, physical activity and sedentary behaviour: a systematic review. Int J Behav Nutr Phys Act.

[14] Redman LM, Gilmore LA, Breaux J, Thomas DM, Elkind-Hirsch K, Stewart T, Hsia DS, Burton J, Apolzan JW, Cain LE, Altazan AD, Ragusa S, Brady H, Davis A, Tilford JM, Sutton EF, Martin CK (2017). Effectiveness of SmartMoms, a Novel eHealth Intervention for Management of Gestational Weight Gain: Randomized Controlled Pilot Trial. JMIR Mhealth Uhealth.

[15] Rhodes A, Smith AD, Chadwick P, Croker H, Llewellyn CH (2020). Exclusively Digital Health Interventions Targeting Diet, Physical Activity, and Weight Gain in Pregnant Women: Systematic Review and Meta-Analysis. JMIR Mhealth Uhealth.

[16] Herring SJ, Cruice JF, Bennett GG, Rose MZ, Davey A, Foster GD (2016). Preventing excessive gestational weight gain among African American women: A randomized clinical trial. Obesity (Silver Spring).

[17] Pollak KI, Alexander SC, Bennett G, Lyna P, Coffman CJ, Bilheimer A, Farrell D, Bodner ME, Swamy GK, Østbye T (2014). Weight-related SMS texts promoting appropriate pregnancy weight gain: a pilot study. Patient Educ Couns.

[18] Willcox JC, Wilkinson SA, Lappas M, Ball K, Crawford D, McCarthy EA, Fjeldsoe B, Whittaker R, Maddison R, Campbell KJ (2017). A mobile health intervention promoting healthy gestational weight gain for women entering pregnancy at a high body mass index: the txt4two pilot randomised controlled trial. BJOG.

[19] Olson CM, Groth SW, Graham ML, Reschke JE, Strawderman MS, Fernandez ID (2018). The effectiveness of an online intervention in preventing excessive gestational weight gain: the e-moms roc randomized controlled trial. BMC Pregnancy Childbirth.

[20] Graham ML, Strawderman MS, Demment M, Olson CM (2017). Does Usage of an eHealth Intervention Reduce the Risk of Excessive Gestational Weight Gain? Secondary Analysis From a Randomized Controlled Trial. J Med Internet Res.

[21] Kennelly MA, Ainscough K, Lindsay KL, OʼSullivan E, Gibney ER, McCarthy M, Segurado R, DeVito G, Maguire O, Smith T, Hatunic M, McAuliffe FM (2018). Pregnancy Exercise and Nutrition With Smartphone Application Support: A Randomized Controlled Trial. Obstet Gynecol.

[22] Ainscough KM, O'Brien EC, Lindsay KL, Kennelly MA, O'Sullivan EJ, O'Brien OA, McCarthy M, De Vito G, McAuliffe FM (2020). Nutrition, Behavior Change and Physical Activity Outcomes From the PEARS RCT-An mHealth-Supported, Lifestyle Intervention Among Pregnant Women With Overweight and Obesity. Front Endocrinol (Lausanne).

[23] Henriksson P, Sandborg J, Blomberg M, Alexandrou C, Maddison R, Silfvernagel K, Henriksson H, Leppänen MH, Migueles JH, Widman L, Thomas K, Trolle Lagerros Y, Löf M (2019). A Smartphone App to Promote Healthy Weight Gain, Diet, and Physical Activity During Pregnancy (HealthyMoms): Protocol for a Randomized Controlled Trial. JMIR Res Protoc.

[24] Eysenbach G, CONSORT-EHEALTH Group (2011). CONSORT-EHEALTH: improving and standardizing evaluation reports of Web-based and mobile health interventions. J Med Internet Res.

[25] Nordic Council of Ministers (2012). Nordic Nutrition Recommendations 2012.

[26] Josefsson A, Haakstad LA, Bö K (2016). FYSS-kapitel FYSISK AKTIVITET VID GRAVIDITET. Rekommendationer om fysisk aktivitet vid graviditet.

[27] Bandura A (1989). Human agency in social cognitive theory. Am Psychol.

[28] Michie S, Richardson M, Johnston M, Abraham C, Francis J, Hardeman W, Eccles MP, Cane J, Wood CE (2013). The behavior change technique taxonomy (v1) of 93 hierarchically clustered techniques: building an international consensus for the reporting of behavior change interventions. Ann Behav Med.

[29] Samdal GB, Eide GE, Barth T, Williams G, Meland E (2017). Effective behaviour change techniques for physical activity and healthy eating in overweight and obese adults; systematic review and meta-regression analyses. Int J Behav Nutr Phys Act.

[30] Soltani H, Arden MA, Duxbury AMS, Fair FJ (2016). An Analysis of Behaviour Change Techniques Used in a Sample of Gestational Weight Management Trials. J Pregnancy.

[31] Rasmussen KM, Yaktine AL (2009). Weight Gain During Pregnancy: Reexamining the Guidelines.

[32] Ruiz JR, Perales M, Pelaez M, Lopez C, Lucia A, Barakat R (2013). Supervised exercise-based intervention to prevent excessive gestational weight gain: a randomized controlled trial. Mayo Clin Proc.

[33] Henriksson P, Löf M, Forsum E (2013). Assessment and prediction of thoracic gas volume in pregnant women: an evaluation in relation to body composition assessment using air displacement plethysmography. Br J Nutr.

[34] van Raaij JM, Peek ME, Vermaat-Miedema SH, Schonk CM, Hautvast JG (1988). New equations for estimating body fat mass in pregnancy from body density or total body water. Am J Clin Nutr.

[35] Moraeus L, Warensjö Lemming E, Hursti UK, Arnemo M, Sipinen JP, Lindroos A (2018). Riksmaten Adolescents 2016-17: A national dietary survey in Sweden - design, methods, and participation. Food Nutr Res.

[36] Moraeus L, Lindroos AK, Warensjö Lemming E, Mattisson I (2020). Diet diversity score and healthy eating index in relation to diet quality and socio-demographic factors: results from a cross-sectional national dietary survey of Swedish adolescents. Public Health Nutr.

[37] Hildebrand M, van Hees VT, Hansen BH, Ekelund U (2014). Age group comparability of raw accelerometer output from wrist- and hip-worn monitors. Med Sci Sports Exerc.

[38] Migueles JH, Cadenas-Sanchez C, Ekelund U, Delisle Nyström C, Mora-Gonzalez J, Löf M, Labayen I, Ruiz JR, Ortega FB (2017). Accelerometer Data Collection and Processing Criteria to Assess Physical Activity and Other Outcomes: A Systematic Review and Practical Considerations. Sports Med.

[39] R [Software].

[40] Migueles JH, Rowlands A, Huber F, Sabia S, van Hees V (2019). GGIR: A Research Community–Driven Open Source R Package for Generating Physical Activity and Sleep Outcomes From Multi-Day Raw Accelerometer Data. J Meas Phys Behav.

[41] Matthews DR, Hosker JP, Rudenski AS, Naylor BA, Treacher DF, Turner RC (1985). Homeostasis model assessment: insulin resistance and beta-cell function from fasting plasma glucose and insulin concentrations in man. Diabetologia.

[42] White IR, Royston P, Wood AM (2011). Multiple imputation using chained equations: Issues and guidance for practice. Stat Med.

[43] Rubin DB (1987). Multiple Imputation for Nonresponse in Surveys.

[44] Vickers AJ, Altman DG (2001). Statistics notes: Analysing controlled trials with baseline and follow up measurements. BMJ.

[45] Ek A, Alexandrou C, Söderström E, Bergman P, Delisle Nyström C, Direito A, Eriksson U, Henriksson P, Maddison R, Trolle Lagerros Y, Bendtsen M, Löf M (2020). Effectiveness of a 3-Month Mobile Phone-Based Behavior Change Program on Active Transportation and Physical Activity in Adults: Randomized Controlled Trial. JMIR Mhealth Uhealth.

[46] Bendtsen M (2018). A Gentle Introduction to the Comparison Between Null Hypothesis Testing and Bayesian Analysis: Reanalysis of Two Randomized Controlled Trials. J Med Internet Res.

[47] Bendtsen M (2019). Electronic Screening for Alcohol Use and Brief Intervention by Email for University Students: Reanalysis of Findings From a Randomized Controlled Trial Using a Bayesian Framework. J Med Internet Res.

[48] Bendtsen M (2019). An Electronic Screening and Brief Intervention for Hazardous and Harmful Drinking Among Swedish University Students: Reanalysis of Findings From a Randomized Controlled Trial Using a Bayesian Framework. J Med Internet Res.

[49] Li L, Aris IM, Han WM, Tan KH (2019). A Promising Food-Coaching Intervention Program to Achieve Optimal Gestational Weight Gain in Overweight and Obese Pregnant Women: Pilot Randomized Controlled Trial of a Smartphone App. JMIR Form Res.

[50] Hayman M, Reaburn P, Browne M, Vandelanotte C, Alley S, Short CE (2017). Feasibility, acceptability and efficacy of a web-based computer-tailored physical activity intervention for pregnant women - the Fit4Two randomised controlled trial. BMC Pregnancy Childbirth.

[51] Choi J, Lee JH, Vittinghoff E, Fukuoka Y (2016). mHealth Physical Activity Intervention: A Randomized Pilot Study in Physically Inactive Pregnant Women. Matern Child Health J.

[52] Walker R, Bennett C, Blumfield M, Gwini S, Ma J, Wang F, Wan Y, Truby H (2018). Attenuating Pregnancy Weight Gain-What Works and Why: A Systematic Review and Meta-Analysis. Nutrients.

[53] Currie S, Sinclair M, Murphy MH, Madden E, Dunwoody L, Liddle D (2013). Reducing the decline in physical activity during pregnancy: a systematic review of behaviour change interventions. PLoS One.

[54] Statistics Sweden Population 16-74 years of age by region, highest level of education, age and sex. Year 1985-2019.

[55] Burrows TL, Ho YY, Rollo ME, Collins CE (2019). Validity of Dietary Assessment Methods When Compared to the Method of Doubly Labeled Water: A Systematic Review in Adults. Front Endocrinol (Lausanne).

[56] Bendtsen M, Bendtsen P, Henriksson H, Henriksson P, Müssener U, Thomas K, Löf M (2020). The Mobile Health Multiple Lifestyle Behavior Interventions Across the Lifespan (MoBILE) Research Program: Protocol for Development, Evaluation, and Implementation. JMIR Res Protoc.

[57] Sandborg J, Henriksson P, Larsen E, Lindqvist AK, Rutberg S, Söderström E, Maddison R, Löf M (2021). Participants’ Engagement and Satisfaction With a Smartphone App Intended to Support Healthy Weight Gain, Diet, and Physical Activity During Pregnancy: Qualitative Study Within the HealthyMoms Trial. JMIR mHealth uHealth.

