# Supplementary material for: Effectiveness of a Smartphone App to Promote Healthy Weight Gain, Diet, and Physical Activity During Pregnancy (HealthyMoms): Randomized Controlled Trial
Source: JMIR Mhealth Uhealth. 2021 Mar 11;9(3):e26091. doi: 10.2196/26091 (PMC7995071; doi:10.2196/26091)
Supplement: Multimedia Appendix 2 [file mhealth_v9i3e26091_app2.pdf]

Table S1. Additional self-reported data on app usage and satisfaction in the intervention group (n=134) at the follow-up measurement. Participants responded to the following statements with the six alternatives shown.

	**Strongly disagree**	**Agree to a small extent**	**Agree to some extent**	**Strongly agree**	**Fully agree**	**Do not know**
	n (%)					
The information on how to use the app was sufficient to get started	0 (0)	0 (0)	0 (0)	15 (11.2)	118 (88.1)	1 (0.7)
The amount of time spent on the app has been enough	1 (0.8)	3 (2.3)	10 (7.5)	39 (29.3)	74 (55.6)	6 (4.5)
I have forgotten to use the app	44 (32.8)	30 (22.4)	38 (28.4)	15 (11.2)	7 (5.2)	0 (0)
I think the app has been boring to use	72 (53.7)	29 (21.6)	17 (12.7)	9 (6.7)	5 (3.7)	2 (1.5)
The app had functions that made it interesting to use	2 (1.5)	4 (3.0)	29 (21.6)	58 (43.3)	39 (29.1)	2 (1.5)
I think the theme texts were good	0 (0)	7 (5.2)	19 (14.2)	55 (41.0)	47 (35.1)	6 (4.5)
It was easy to learn the functions of the app	0 (0)	2 (1.5)	2 (1.5)	32 (23.9)	96 (71.6)	2 (1.5)
It was easy to register my physical activity in the app	4 (3.0)	5 (3.7)	10 (7.5)	28 (20.9)	50 (37.3)	37 (27.6)
It was easy to register my diet in the app	6 (4.5)	12 (9.0)	18 (13.4)	25 (18.7)	31 (23.1)	42 (31.3)
It was easy to register my weight gain in the app	1 (0.7)	1 (0.7)	4 (3.0)	13 (9.7)	101 (75.4)	14 (10.4)
It was time consuming to register in the app	55 (41.0)	34 (25.4)	19 (14.2)	7 (5.2)	4 (3.0)	15 (11.2)
I think the pregnancy calendar was good	0 (0)	4 (3.0)	14 (10.4)	30 (22.4)	67 (50.0)	19 (14.2)
I perceived the factual content in the app as correct	1 (0.7)	1 (0.7)	4 (3.0)	41 (30.6)	82 (61.2)	5 (3.7)
It was easy to navigate in the app	2 (1.5)	3 (2.2)	12 (9.0)	39 (29.1)	76 (56.7)	2 (1.5)
I found the appearance of the app as appealing	3 (2.2)	9 (6.7)	26 (19.4)	37 (27.6)	57 (42.5)	2 (1.5)
I experienced technical issues with the app	114 (85.1)	9 (6.7)	5 (3.7)	1 (0.7)	2 (1.5)	3 (2.2)
The app consumed a lot of battery power	69 (51.5)	2 (1.5)	3 (2.2)	0 (0)	0 (0)	60 (44.8)
I read most of the messages I received through the app	9 (6.7)	8 (6.0)	21 (15.7)	42 (31.3)	50 (37.3)	4 (3.0)
The number of messages were too many	61 (45.5)	23 (17.2)	26 (19.4)	10 (7.5)	3 (2.2)	11 (8.2)
The messages gave me valuable tips and information	5 (3.7)	20 (14.9)	42 (31.3)	35 (26.1)	21 (15.7)	11 (8.2)
I usually use similar apps	42 (31.3)	25 (18.7)	23 (17.2)	26 (19.4)	17 (12.7)	1 (0.7)
