# Supplementary material for: Effectiveness of a Smartphone App to Promote Healthy Weight Gain, Diet, and Physical Activity During Pregnancy (HealthyMoms): Randomized Controlled Trial
Source: JMIR Mhealth Uhealth. 2021 Mar 11;9(3):e26091. doi: 10.2196/26091 (PMC7995071; doi:10.2196/26091)
Supplement: Multimedia Appendix 3 [file mhealth_v9i3e26091_app3.pdf]

Table S2. Intervention effect on the components in the Swedish Healthy Eating Index.

Outcome	Descriptive data		Intervention effect using regression analysis[a]			
	Mean (SD)		Imputed data analysis (n=305)		Complete cases analysis (n=266)	
	Intervention	Control	Coefficient (95% CI)	P-value	Coefficient (95% CI)	P-value
**Fruit & vegetables (g/day)**						
Baseline, n=302[b]	448 (184)	465 (194)	29.3	.17	26.8	.22
Follow-up, n=269[b]	434 (189)	428 (223)	(-12.2; 70.8)		(-15.8; 69.5)	
**Fibre (g/MJ)[c]**						
Baseline, n=302[b]	2.70 (0.72)	2.76 (0.85)	0.03	.66	0.03	.66
Follow-up, n=269[b]	2.59 (0.64)	2.65 (0.92)	(-0.12; 0.19)		(-0.12; 0.19)	
**Wholegrain (g/MJ)[c]**						
Baseline, n=302[b]	4.56 (2.85)	4.57 (2.83)	0.20	.56	0.20	.58
Follow-up, n=269[b]	4.74 (2.85)	4.58 (3.02)	(-0.48; 0.89)		(-0.49; 0.89)	
**Fish & shellfish (g/day)**						
Baseline, n=302[b]	28.2 (32.6)	37.4 (37.7)	2.8	.47	2.3	.56
Follow-up, n=269[b]	27.0 (29.7)	25.5 (33.6)	(-4.8; 10.4)		(-5.4; 10.0)	
**PUFA (E%)**						
Baseline, n=302[b]	5.28 (1.54)	5.69 (1.56)	0.06	.74	0.09	.62
Follow-up, n=269[b]	5.27 (1.65)	5.44 (1.74)	(-0.30; 0.42)		(-0.28; 0.47)	
**MUFA (E%)**						
Baseline, n=302[b]	14.6 (3.6)	15.4 (3.1)	-0.06	.87	-0.06	.87
Follow-up, n=269[b]	14.5 (2.9)	14.8 (3.1)	(-0.75; 0.63)		(-0.76; 0.64)	
**SFA (E%)**						
Baseline, n=302[b]	15.6 (3.1)	15.5 (3.5)	0.27	.42	0.19	.56
Follow-up, n=269[b]	15.7 (2.7)	15.4 (3.3)	(-0.39; 0.93)		(-0.47; 0.85)	
**Red meat (g/week)[d]**						
Baseline, n=302[b]	472 (360)	456 (357)	-86.5	.027	-86.8	.029
Follow-up, n=269[b]	431 (267)	503 (391)	(-163.2; -9.90)		(-164.5; -9.20)	
**Sucrose (E%)**						
Baseline, n=302[b]	8.28 (3.80)	7.65 (2.73)	-0.18	.66	-0.19	.64
Follow-up, n=269[b]	8.61 (3.67)	8.56 (3.38)	(-1.00; 0.63)		(-1.01; 0.63)	

SD: standard deviation, CI: confidence interval, E%: percentage of total daily energy intake, MJ: mega joule, PUFA: poly-unsaturated fatty acids, MUFA: mono-unsaturated fatty acids, SFA: saturated fatty acids

[a] Regression analysis of follow-up measure of dietary outcome on group allocation. The coefficient is interpreted as the estimated effect of the intervention compared to the control adjusted for baseline value of the dietary outcome, BMI category (underweight and normal weight vs overweight and obesity), and parity (0 vs 1 or more), and educational attainment (university degree vs no university degree).

[b] Baseline, n=302 (151 intervention and 151 control); Follow-up, n=269 (135 intervention and 134 control).

[c] Gram per MJ of total daily energy intake

[d] In order to be comparable with international and national recommendations, average red meat intake was expressed as gram/week by multiplying average daily intake with seven
